# Speicheldrüsenkarzinome – ein aktueller Überblick: Fortschritte in der molekularen Typisierung Teil II

**DOI:** 10.1007/s00292-022-01171-4

**Published:** 2023-01-09

**Authors:** Niels J. Rupp, Sandra N. Freiberger

**Affiliations:** 1 Institut für Pathologie und Molekularpathologie, Universitätsspital Zürich, Zürich, Schweiz Schmelzbergstr. 12, 8091; 2 Universität Zürich, Zürich, Schweiz

**Keywords:** Molekulare Pathologie, Morphologische und mikroskopische Befunde, Genfusion, ErbB-2-Rezeptor, Androgenrezeptor, Molecular pathology, Morphological and microscopic findings, Gene fusion, ErbB‑2 receptor, Androgene receptor

## Abstract

Der zweite Teil des Artikels widmet sich den molekularen Charakteristiken des epithelial-myoepithelialen Karzinoms, des polymorphen Adenokarzinoms, des myoepithelialen Karzinoms, des Basalzelladenokarzinoms sowie des Speichelgangkarzinoms. Zusätzlich werden die neu aufgekommenen Entitäten des muzinösen Adenokarzinoms, des sklerosierenden mikrozystischen Adenokarzinoms und des mikrosekretorischen Adenokarzinoms zusammengefasst. Auch bei den meisten dieser Entitäten kann der molekulare Genotyp diagnostisch sehr hilfreich sein. Eine Überexpression des Androgenrezeptors und/oder von „human epidermal growth factor receptor 2“ (HER2)/neu kann dabei im geeigneten histopathologischen Kontext nicht nur zur Diagnoseuntermauerung eines Speichelgangkarzinoms dienen, sondern potenziell auch gezielt therapeutisch angegangen werden.

## Abstract (en)

The second part of the article is devoted to the molecular characteristics of epithelial–myoepithelial carcinoma, polymorphous adenocarcinoma, myoepithelial carcinoma, basal cell adenocarcinoma, and salivary duct carcinoma. In addition, the new entities mucinous adenocarcinoma, sclerosing microcystic adenocarcinoma, and microsecretory adenocarcinoma are summarized. The molecular genotype can also be very helpful in diagnosing most of these entities. In this regard, overexpression of the androgen receptor and/or human epidermal growth factor receptor 2 (HER2)/neu can support diagnosis in the appropriate histopathologic context and may serve as a potential target for therapy in salivary duct carcinoma.

## Lernziele

Nach Lektüre dieses Beitrags ...

- können Sie weitere relevante molekulare Alterationen in Speicheldrüsenkarzinomen benennen;
- kennen Sie das morphologische und molekulare Spektrum des polymorphen Adenokarzinoms;
- sind Ihnen neu definierte Entitäten des Speicheldrüsenkarzinoms, wie das mikrosekretorische Adenokarzinom, bekannt.

## Hintergrund

Im 2. Teil dieser Arbeit wird der Überblick über den aktuellen Stand der morpho-molekularen Typisierung von Speicheldrüsenkarzinomen auf weitere etablierte Entitäten erweitert. Außerdem werden 3 neu definierte Entitäten von Speicheldrüsenkarzinomen umschrieben.

## Molekulare Eigenschaften etablierter Entitäten

### Epithelial-myoepitheliales Karzinom

**Figure 1:**
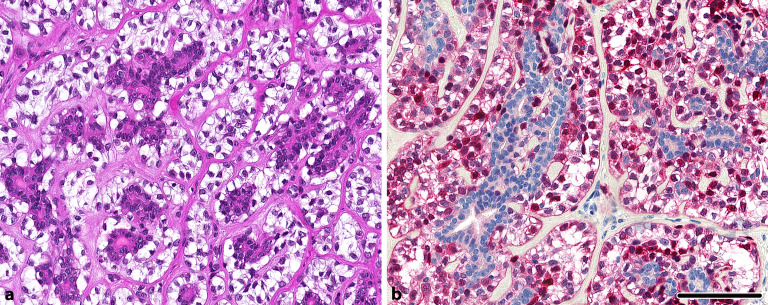

Das epithelial-myoepitheliale Karzinom (EMC) ist in seiner klassischen Differenzierung zumeist klar zu erkennen (Abb. 1). Diagnostische Schwierigkeiten bereitet vor allem die gelegentlich fehlende Invasion mit scharfer Umgrenzung der Läsion. Die EMC zeigen morphologisch und auch immunhistochemisch deutlich belegbar eine **biphasische Differenzierung**

biphasische Differenzierung

mit äußerer myoepithelialer und innerer epithelialer Schicht. Diagnostisch insbesondere herausfordernd sind **verschiedene Varianten**

verschiedene Varianten

wie die doppelklarzellige, sebaziöse, basaloide, apokrine oder auch (solid-)onkozytäre Variante [1, 2]. Kürzlich konnte in einer großen Arbeit gezeigt werden, dass EMC rekurrente aktivierende ***HRAS*-Mutationen**

HRAS-Mutationen

(zumeist Q61R), gelegentlich auch in Zusammenschau mit *PIK3CA*- oder *AKT1*-Mutationen zeigen (Tab. 1; [1]). Diese können zusammen mit der Biphasizität als molekularer Beleg verwendet werden, insbesondere da *HRAS*-Mutationen in Speicheldrüsentumoren insgesamt nur sehr selten in benignen Tumoren, wie z. B. dem Sialadenoma papilliferum, beschrieben sind [3]. Eine **Immunhistochemie**

Immunhistochemie

mit dem mutationsspezifischen RAS(Q61R)-Antikörper kann dabei diagnostisch hilfreich sein, da er neben *NRAS* auch an dieser Position mutiertes *HRAS* identifizieren kann. Interessanterweise zeigt sich dabei ein ungewöhnliches Muster mit membranöser Expression in den myoepithelialen Zellen bei typischerweise Aussparungen in den epithelialen Zellen ([4]; Abb. 1). Im Gegensatz dazu zeigen EMC ex pleomorphem Adenom (ex PA) typischerweise keine *HRAS*-Mutation, sondern **Translokationen**

Translokationen

des *PLAG1*- oder *HMGA2*-Gens [5].

**Table 1:** 

Entität	Molekulare Alteration	Quelle
*Epithelial-myoepitheliales Karzinom*	*HRAS*-, *PIK3CA* und *AKT1*-Mutationen	[1]
*Polymorphes Adenokarzinom*		
Konventioneller Subtyp	*PRKD1*(E710D)-Mutation	[6, 7]
Kribriformer Subtyp	*PRKD1-, PRKD2-* oder *PRKD3*-Genfusion	[6, 7]
*Myoepitheliales Karzinom*	*PLAG1*-Genfusionen; insbesondere *TGFBR3-PLAG1*	[8, 9]
*Speichelgangkarzinom*	Androgenrezeptorüberexpression, HER2/neu-Überexpression; *TP53-, HRAS-* und *PIK3CA*-Mutationen	[10]
*Muzinöses Adenokarzinom*	*AKT1*(E17K)-Mutation, *TP53*-Mutationen	[11]
*Sklerosierendes mikrozystisches Adenokarzinom*	Keine rekurrenten Alterationen bekannt	–
*Mikrosekretorisches Adenokarzinom*	*MEF2C-SS18* Genfusion	[12]

#### Merke

Die membranöse Expression des RAS(Q61R)-mutationsspezifischen Antikörpers kann auf eine entsprechende *HRAS*-Mutation hinweisen und bei geeigneter Morphologie die Diagnose eines epithelial-myoepithelialen Karzinoms unterstützen.

### Polymorphes Adenokarzinom

Das polymorphe Adenokarzinom ist durch einen **monomorphen Zelltyp**

monomorphen Zelltyp

gekennzeichnet, der zahlreiche morphologische Wachstumsformen aufweisen kann. Diese Karzinome präsentieren sich in aller Regel in der Mundhöhle, insbesondere am Gaumen. Es lassen sich 2 morphologisch distinkte Varianten unterscheiden, der klassische oder konventionelle Subtyp und der **kribriforme Subtyp**

kribriforme Subtyp

**Figure 2:**
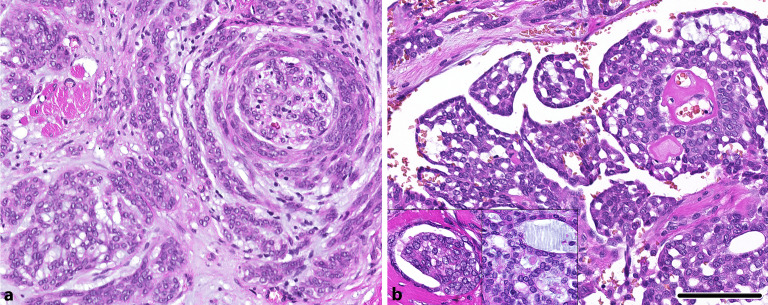

[13]. Darunter findet sich beim konventionellen Subtyp typischerweise ein tubuläres, trabekuläres, mikrozystisches sowie targetoides perineurales Wachstum (Abb. 2). Der kribriforme Subtyp hingegen zeigt einen ähnlichen Zelltyp, der typischerweise jedoch aufgehellte Kerne umfasst, die an ein papilläres Schilddrüsenkarzinom erinnern. Das **Wachstumsmuster**

Wachstumsmuster

dabei zeigt oftmals ein kribriformes, papilläres sowie auch glomeruloides Bild (Abb. 2). Der kribriforme Subtyp, der in der Literatur auch unter dem Begriff des kribriformen Adenokarzinoms der Speicheldrüsen (CASG) zu finden ist, zeigt häufiger Lymphknotenmetastasen. Der Nachweis einer papillären Differenzierung von ≥ 10 % und/oder einer kribriformen Differenzierung von ≥ 30 % wurde dabei als negativer **prognostischer Parameter**

prognostischer Parameter

(„erkrankungsfreies Überleben“) identifiziert [14]. Molekular findet sich im konventionellen Subtyp am häufigsten eine E710D-Punktmutation im ***PRKD1*-Gen**

PRKD1-Gen,

wohingegen der kribriforme Subtyp zumeist Genfusionen im *PRKD1*-, *PRKD2*- oder *PRKD3*-Gen zeigt [6]. In seltenen Fällen finden sich sowohl morphologische Überlappungen ohne eindeutige Subtypzuordnung als auch molekulare Veränderungen, die nicht mit der Morphologie im Einklang stehen (*PRKD1*-Punktmutation vs. *PRKD-*Genfusion; [15]). Der Nachweis einer *PRKD1*-, -*2* oder -*3-*Genfusion ist dabei als Risikofaktor für häufigere **Lymphknotenmetastasen**

Lymphknotenmetastasen

beschrieben [14]. Abschließend sei anzumerken, dass das Spektrum polymorpher Adenokarzinome in sehr seltenen, aber gut dokumentierten Fällen auch in der Parotis zu finden ist [6, 7].

#### Merke

Der Nachweis einer papillären (≥ 10 %)/kribriformen (≥ 30 %) Morphologie sowie einer *PRKD*-Genfusion wurde als negativer prognostischer Parameter bzw. Risikofaktor für häufigere Lymphknotenmetastasen im polymorphen Adenokarzinom identifiziert.

### Myoepitheliales Karzinom

Das myoepitheliale Karzinom gehört zu den diagnostisch herausforderndsten Karzinomen der Speicheldrüse. Diese sind gekennzeichnet durch ein **monophasisches Karzinom**

monophasisches Karzinom

mit morphologisch myoepithelialer Differenzierung (u. a. spindelzellig, plasmazytoid), das typischerweise eine Koexpression von SOX10 und S‑100 aufweist. Die Wachstumsmuster können dabei variabel sein und unter anderem eine retikuläre, trabekuläre und solide Differenzierung zeigen, wobei ein charakteristisches Merkmal peripher **hyperzelluläre Knoten**

hyperzelluläre Knoten

sind, die zum Zentrum hypozellulär werden [13]. Weitere Marker können SMA, Calponin und p63 umfassen; diese finden sich jedoch variabel exprimiert [16]. Insbesondere myoepitheliale Karzinome ex PA, die zu den häufigsten sekundären Karzinomen gehören, werden häufig unterdiagnostiziert [17], wobei als Unterscheidungsmerkmal das **infiltrative Wachstumsmuster**

infiltrative Wachstumsmuster

der myoepithelialen Komponente zu nennen ist. Problematisch ist dabei, dass trotz geringer Atypien diese Karzinome häufiger ein biologisch aggressives Verhalten zeigen [16]. Molekular zeigen myoepitheliale Karzinome einen überlappenden Genotyp mit pleomorphen Adenomen, sodass nicht selten *PLAG1*-Genfusionen mit verschiedenen Fusionspartnern beschrieben sind [8, 18]. In der Regel lässt sich somit aus dem molekularen Profil keine Aussage über die Entität und Dignität ablesen, da z. B. auch Myoepitheliome *PLAG1-*Genfusionen aufweisen können [19]. Insbesondere an bioptischem oder Feinnadelmaterial kann ohne Nachweis einer Invasion keine Unterscheidung zwischen einem Myoepitheliom und einem „low-grade“ myoepithelialen Karzinom getroffen werden. Eine Ausnahme scheint dabei die ***TGFBR3*–*PLAG1*-Fusion**

TGFBR3–PLAG1-Fusion

zu sein, die in einer großen Kohorte ausschließlich in malignen Tumoren (de novo myoepitheliale Karzinome oder myoepitheliale Karzinome ex PA) identifiziert wurde [8]. In einer weiteren Arbeit wurde die gleiche Genfusion in 3 Tumoren im tiefen Parotislappen identifiziert, die morphologische Eigenschaften eines „low-grade“ myoepithelialen Karzinoms aufwiesen [9].

#### Merke

Myoepitheliale Karzinome zeigen häufig Genfusionen des *PLAG1*-Gens.

#### Merke

Myoepitheliale Karzinome werden häufig unterdiagnostiziert und zeigen trotz blander Morphologie oftmals ein biologisch aggressives Verhalten.

### Basalzelladenokarzinom

Das Basalzelladenokarzinom ist insgesamt sehr selten und zeigt eine biphasische epithelial-myoepitheliale Differenzierung mit **basaloidem Zellaspekt**

basaloidem Zellaspekt.

Im Gegensatz zum Basalzelladenom findet sich ein infiltratives Wachstumsmuster, das als einziges sicheres Unterscheidungsmerkmal dient [20]. Molekular finden sich verschiedene Alterationen, darunter ***CYLD*-Mutationen**

CYLD-Mutationen,

selten auch *CTNNB1*-Mutationen [21]. Letztere wiederum finden sich häufig in Basalzelladenomen, sodass davon auszugehen ist, dass sich ein kleiner Teil der Basalzelladenokarzinome über eine Sequenz aus Basalzelladenomen entwickelt; der größte Teil dürfte distinkt de novo entstehen. Sowohl Basalzelladenome als auch Basalzelladenokarzinome können eine nukleäre **β‑Catenin-Reaktivität**

β‑Catenin-Reaktivität

zeigen, sodass damit das Spektrum der Basallzellneoplasien untermauert werden kann. Es kann jedoch keine zuverlässige Aussage über die Dignität getroffen werden [20].

### Speichelgangkarzinom

Das Speichelgangkarzinom ist als relativ häufige Entität anzutreffen und zeigt praktisch ausschließlich eine „high-grade“ Morphologie mit einem entsprechend hohen Risiko von Lymphknoten- und Fernmetastasen. Molekular finden sich neben ***TP53*-Mutationen**

TP53-Mutationen

ebenfalls häufig *HRAS*- und *PIK3CA*-Mutationen [10]. Relevant sind vor allem Alterationen, die zu einer Überexpression von **Androgenrezeptor**

Androgenrezeptor

(AR) und HER2/neu führen, da diese potenziell gezielt therapeutisch angegangen werden können [22]. Insbesondere die AR-Überexpression kann diagnostisch wertvoll sein und als charakteristischer Marker die Diagnose eines Speichelgangkarzinoms erhärten. Dadurch kann auch die Gruppe der nicht anderweitig spezifizierten (Adeno‑)Karzinome (NOS) weiter reduziert werden [23]. Eine aberrante immunhistochemische TP53-Expression (kräftige diffuse Überexpression oder vollständiger Verlust der Expression) als **Surrogatmarker**

Surrogatmarker

für eine Mutation kann die Diagnose unterstützen ist jedoch naturgemäß weitaus weniger spezifisch als die AR-Überexpression.

#### Merke

Die Androgenrezeptor- und die HER2/neu-Überexpression im Speichelgangkarzinom können diagnostisch und potenziell gezielt therapeutisch genutzt werden.

## Neue morphologisch und molekular definierte Entitäten

### Muzinöses Adenokarzinom

**Figure 3:**
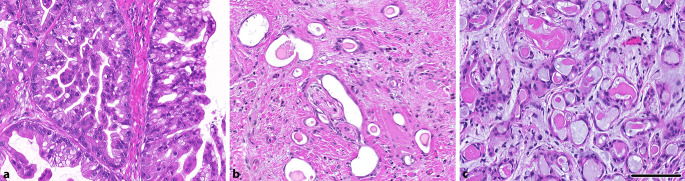

Das muzinöse Adenokarzinom erinnert morphologisch an muzinöse Adenokarzinome des Gastrointestinaltrakts bzw. des pankreatobiliären Trakts. Es zeigt sich typischerweise reichlich **extrazelluläre Schleimbildung**

extrazelluläre Schleimbildung

mit papillärer, trabekulärer sowie selten auch siegelringzelliger Differenzierung ([11]; Abb. 3a). Immunhistochemisch sind die Karzinome typischerweise positiv für CK7 bei Negativität für CK20 und CDX2. Nicht selten sind Metastasen in den regionären Lymphknoten anzutreffen. In den bisher molekular charakterisierten Fällen findet sich sehr häufig (> 90 %) eine rekurrente E17K-Mutation im ***AKT1*-Gen**

AKT1-Gen,

die grundsätzlich auch diagnostisch zur Untermauerung verwendet werden kann [13]. Hierbei sei anzumerken, dass bei den muzinösen Adenokarzinomen zusätzlich zumeist *TP53*-Mutationen vorliegen [11]; im Gegensatz zu den morphologisch ähnlichen Läsionen, für die der Begriff der intraduktalen papillären muzinösen Neoplasie (IPMN) vorgeschlagen wurde [24]. Letztere zeigen ein überlappendes morphologisches Spektrum und ebenfalls *AKT1*-(E17K)-Mutationen, sind aber biologisch als indolent einzustufen. Zur genaueren Einordnung der Verwandtschaft dieser Tumoren werden weitere Daten benötigt [25].

### Sklerosierendes mikrozystisches Adenokarzinom

Das sklerosierende mikrozystische Adenokarzinom ist quasi das Pendant zum mikrozystischen Adnexkarzinom der Haut und hat einen ähnlichen morphologischen Aufbau. In einem sklerosierten Hintergrund zeigen sich äußerst hochdifferenzierte tubuläre Formationen mit biphasischer epithelial-myoepithelialer Differenzierung, die diffus infiltrieren und nicht selten eine **Perineuralscheideninfiltration**

Perineuralscheideninfiltration

zeigen ([26]; Abb. 3b). Bisher sind keine rekurrenten molekularen Alterationen bekannt, lediglich in einem Fall ist eine *CDK11B*-Mutation beschrieben [27].

### Mikrosekretorisches Adenokarzinom

Das kürzlich charakterisierte mikrosekretorische Adenokarzinom ist ein Karzinom der Speicheldrüsen und findet sich typischerweise in der Mundhöhle. Die Morphologie zeigt kleine, irreguläre Drüsen, die an „intercalated ducts“ erinnern und mit einem bläulichen Material gefüllt sind (Abb. 3c). Immunhistochemisch zeigt sich eine Expression von S‑100 und SOX10 [28] sowie eine Expression von p63 bei Negativität für p40. Molekular findet sich eine rekurrente ***MEF2C*-*SS18-*Genfusion**

*MEF2C*-*SS18*-Genfusion,

die mittels Fluoreszenz-in-situ-Hybridisierung (FISH) oder RNA-Sequenzierung nachgewiesen werden kann [29]. Die **Differenzialdiagnose**

Differenzialdiagnose

umfasst insbesondere ein adenoid-zystisches Karzinom, ein polymorphes Adenokarzinom und ein Mukoepidermoidkarzinom. Eine Unterscheidung ist hierbei insbesondere klinisch von Relevanz, da in den bisher publizierten Fällen weder ein Rezidiv noch Metastasen beschrieben worden sind [12].

#### Merke

Das mikrosekretorische Karzinom scheint biologisch sehr indolent, sodass eine Abgrenzung von anderen morphologisch ähnlichen Entitäten von besonderer Relevanz ist.

## Fazit für die Praxis

- Die RAS(Q61R)-Immunhistochemie kann als Surrogatmarker auf eine entsprechende *HRAS*-Mutation hinweisen.
- Die* PLAG1*-Genfusionen sind als Ausnahme insgesamt wenig spezifisch und können in benignen und malignen Neoplasien gefunden werden.
- Die korrekte Diagnose des mikrosekretorischen Adenokarzinoms ist im Hinblick auf das offensichtlich wenig aggressive Verhalten von Relevanz.
- Androgenrezeptor*-* und/oder HER2/neu-Überexpression können potenziell als therapeutischer Angriffspunkt dienen.

## CME-Fragebogen

Sie diagnostizieren ein epithelial-myoepitheliales Karzinom. Welche molekulare Veränderung erwarten Sie am wahrscheinlichsten?

*TP53*-Genmutation

*NCOA4-RET*-Genfusion

*HRAS*-Genmutation

*TRIM33-RET*-Genfusion

*MEF2C-SS18*-Genfusion

Sie erhalten eine grenzwertig repräsentative Biopsie vom harten Gaumen. Es zeigt sich ein fokaler myxoider Hintergrund mit wenigen, relativ monomorphen basaloiden Zellverbänden. Sie stellen korrekt die Differenzialdiagnose zwischen einem polymorphen Adenokarzinom und einem pleomorphen Adenom. Welche molekularen Veränderungen können Ihnen zur weiteren Einordnung helfen?

*HRAS*-Genmutation vs. *PLAG1*-Genfusion

*PRKD1*-Genmutation* vs. PLAG1*-Genfusion

*EWSR1*-Genfusion* vs. NR4A3*-Hochregulierung

*PRKD1*-Genmutation* vs. RET*-Genfusion

*SS18*-Genfusion vs. *NTRK*-Genfusion

Sie erhalten eine Biopsie aus der oralen Mukosa und stellen die Verdachtsdiagnose eines mikrosekretorischen Adenokarzinoms. Mit welcher Untersuchung kann ein solches molekular untermauert und von morphologisch ähnlichen Entitäten abgegrenzt werden?

*SS18*-Fluoreszenz-in-situ-Hybridisierung(FISH)/RNA-Sequenzierung

*MYB*-FISH/RNA-Sequenzierung

*MAML2*-FISH/RNA-Sequenzierung

*RET*-FISH/RNA-Sequenzierung

*ETV6*-FISH/RNA-Sequenzierung

Bei der gemeinsamen Befundung sehen Sie eine basaloide Speicheldrüsenneoplasie der Parotis mit biphasischem Aufbau und herdförmiger, deutlicher nukleärer β‑Catenin-Expression. Was raten Sie der vorstellenden Kollegin/dem vorstellenden Kollegen?

Es handelt sich um ein Basalzelladenom.

Es handelt sich um ein Basalzelladenokarzinom.

Es handelt sich um einen Tumor aus dem Spektrum der Basalzellneoplasien, zur Dignitätsbeurteilung (Basalzelladenom vs. Basalzelladenokarzinom) muss nach einer Invasion gesucht werden.

Es handelt sich um ein zellreiches pleomorphes Adenom.

Es handelt sich um ein sekretorisches Karzinom.

Welches ist das typische Merkmal von myoepithelialen Karzinomen?

Biphasische Differenzierung

Knotiges Wachstum mit hyperzellulärer Peripherie und hypozellulärem Zentrum

Fehlende Perineuralscheideninfiltration

Onkozytäre Differenzierung

Nachweis intratumoraler Zymogengranula

Welche Konstellation findet sich im Speichelgangkarzinom am häufigsten?

Androgenrezeptorüberexpression und HER2/neu-Überexpression

*HRAS-* und *PRKD1*-Genmutation

*ETV6-NTRK3- und NCOA4-RET-*Genfusion

*SS18-MEF2C-*Genfusion* und PIK3CA-*Genmutation

*CRTC1-MAML2-*Genfusion* und HRAS-*Genmutation

Was trifft über das sklerosierende mikrozystische Adenokarzinom zu?

Es ist molekular klar definiert

Es zeigt eine große morphologische Ähnlichkeit zum mikrozystischen Adnexkarzinom der Haut

Es metastasiert häufig in die Lymphknoten

Es zeigt praktisch nie eine Perineuralscheideninfiltration

Typisch sind „high-grade“ Kernatypien

Sie sehen histologisch einen relativ scharf abgegrenzten chrondromyxoiden, zellarmen, fibrosierten Knoten, um den sich eine monomorphe und monophasische Population aus zytologisch blanden, jedoch infiltrativ wachsenden Zellen zeigt. Molekular findet sich eine *PLAG1*-Genfusion. Welches ist die wahrscheinlichste Diagnose?

Sekretorisches Karzinom

Myoepitheliales Karzinom ex pleomorphem Adenom

Myoepitheliom

Adenoid-zystisches Karzinom ex pleomorphem Adenom

Epithelial-myoepitheliales Karzinom ex pleomorphem Adenom

Welche ist die charakteristische molekulare Alteration des muzinösen Adenokarzinoms der Speicheldrüsen?

*AKT1*-(E17K)-Genmutation

*EWSR1-ATF1*-Genfusion

*MEF2C-SS18*-Genfusion

*HRAS*-Genmutation

*PRKD1*-Genmutation

Welche Eigenschaft beim Spektrum der polymophen Adenokarzinome ist mit einem erhöhten Risiko für Lymphknotenmetastasen assoziiert?

*MEF2C-SS18*-Genfusion

*PRKD1*-*, -2*-*, oder -3*-Genfusion

*EWSR1-ATF1*-Genfusion

PRKD1-Genmutation

*CRTC1-MAML2*-Genfusion
